# PHLD Class Proteins: A Family of New Players in the p53 Network

**DOI:** 10.3390/ijms21103543

**Published:** 2020-05-17

**Authors:** Taylor T. Fuselier, Hua Lu

**Affiliations:** 1 Department of Biochemistry and Molecular Biology, Tulane University School of Medicine, New Orleans, LA 70112, USA; tfuselie@tualne.edu; 2 Tulane Cancer Center, Tulane University School of Medicine, New Orleans, LA 70112, USA

**Keywords:** PHLDA, PHLDB, p53, AKT signaling, cancer, metabolism

## Abstract

The Pleckstrin Homology-like Domain (PHLD) class of proteins are multifunctional proteins. The class is comprised of two families of proteins, PHLDA and PHLDB, each with 3 members. All members of the families possess a pleckstrin homology (PH) domain. Though identified nearly 30 years ago, this class of proteins remains understudied with PHLDA family members receiving most of the research attention. Recent studies have also begun to reveal the functions of the PHLDB family proteins in regulation of p53 and AKT signaling pathways important for cancer and metabolism. This review will discuss current research and offer some prospects on the possible roles of both families in cancer and metabolism.

## 1. Introduction

Pleckstrin homology (PH) protein domains are highly conserved throughout eukaryotes and are readily found in the human proteome. Proteins possessing PH domains were originally characterized from the region of the pleckstrin protein that binds with high in vitro specificity to the phosphorylated head group of phosphatidylinositol 4,5-bisphosphate (PtdI(4,5)P_2_) lipids [1], however, less than 10% of identified proteins possessing shared sequence homology for PH domains bind with high specificity to phosphorylated phosphatidylinositol lipids [2]. Rather, those PH domain containing proteins originally shown to bind to one species of phosphorylated phosphatidylinositol (PtdI(4,5)P_2_) lipids bind to several species of phosphorylated PtdI lipids with the caveat that PtdI lipids have adjacent phosphates present on the inositol head group (i.e. PtdI(1,4,5)P_3_, PtdI(3,4,5)P_3_, etc.) [2,3]. 

**Figure 1:**
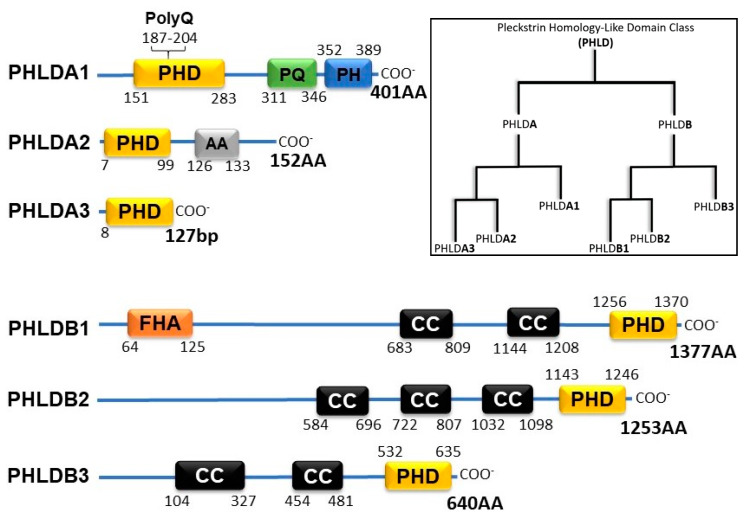
Schematic of PHLD class proteins and phylogenetic tree adopted from constraint-based alignment tool (COBALT, NIH). PHD (Pleckstrin Homology Domain); PolyQ (Polyglutamine); PQ (Proline Glutamine Repeat); PH (Proline Histidine Repeat); AA (Alanine Repeats); FHA (Forkhead-associated Domain); CC (Coiled-coil Domain).

The Pleckstrin Homology-like Domain (PHLD) class of proteins are arranged in two separate families, PHLDA and PHLDB. Each of the families is composed of 3 members: PHLDA1, PHLDA2, PHDLA3, PHLDB1, PHLDB2, and PHLDB3. As their name suggests, all PHLD class proteins possess a PH domain shown to bind to phosphorylated Ptdl lipids. The two families of PHLD proteins differ from one another by position of their PH domain either on the N-terminus or the C-terminus, longer sequence lengths, and coiled-coil domains (Figure 1). PHLDA1 has a unique split PH domain, interrupted by a polyglutamine region (PolyQ), that no other class members possess. In addition, PHLDB1 has a forkhead-associated (FHA) domain that no other class member possesses and enables PHLDB1 to bind to phosphorylated threonine and tyrosine residues [4].

PHLDA family members (in particular, PHLDA1) are disproportionately studied relative to their PHLDB family members. PHLDA1, originally *TDAG51,* was the first discovered PHLDA family member thought to be necessary for peripheral T-cell apoptosis after Fas activation [5]. The “Pleckstrin homology-like domain” family was formally named after the addition of two other PHLDA family members, PHLDA2 (formerly Ipl) and PHLDA3 (formerly Tih1), based on sequence homology, similar chromosomal location, and similar genetic imprinting patterns [6]. PHLDB family members, PHLDB1 (formerly LL5α) and PHLDB2 (formerly LL5β), were identified from amino acid sequence homology to known proteins that possessed PH domains previously characterized by their in vitro affinities to PtdI(3,4,5)P_3_, PtdI(3,4)P_2_ or PtdI(4,5)P_2_ [3]. The final member of the PHLDB family, PHLDB3, is by far the most understudied family member with one dedicated publication originating from our lab. Over the past decade, several studies of these two families have unraveled their roles in regulation of metabolism and cancer development, particularly involving p53 and AKT pathways. However, there are few review articles documenting the progress of research. Thus, in this essay, we will discuss the most recent progress with some prospects on the p53-dependent and independent roles of these families in cancer and metabolism, but apologize to those whose publications on these families might be overlooked.

## 2. PHLD Class Proteins and Cancer

PHLD class of proteins have been linked to both cancer progression and suppression, as summarized in Table 1. PHLDA1 has been shown to act as an oncogene or a tumor-suppressor depending on what tissues are involved as reviewed previously [7]. RNA-seq analysis of osteosarcoma patient samples and cell lines showed increased expression of PHLDA1, which is correlated with poor patient prognosis and highly metastatic cell lines [8]. PHLDA1 expression is increased in patient-derived gliomas and correlated with increased expression of a long non-coding RNA proposed to hybridize with microRNA, miR-194, responsible for silencing PHLDA1 expression [9]. Interestingly, PHLDA1 has multiple glutamine repeats (PolyQ region) within the PH domain sequence. The PolyQ regions are known to be hotspots of genomic instability that can expand or contract during replication and thus alter the stability of translated proteins [10]. It remains to be studied whether PHLDA1 has variable PolyQ polymorphisms leading to altered protein functions like several PolyQ proteins expressed in neurological diseases [7]. On the other hand, the PHLDA family members can function as tumor suppressors by inhibiting AKT activation in breast [11,12], ovarian [11], endometrial [12], and lung [13,14] cancers. The PH domain of the PHLDA family members outcompetes AKT PH domain by binding to phosphorylated PtdI lipids at the cell membrane, resulting in incomplete AKT activation via phosphorylation of AKT at serine-473 [11,12,13,14]. Fearon et al. recently proposed a role for PHLDA1 to mitigate resistance of reoccurring cancers post-receptor tyrosine kinase (RTK) inhibitor therapies [12]. Fibroblast growth factor receptor (FGFR), a RTK, contributes to oncogenic signaling pathways, yet inhibition of this signaling pathway with chemotherapeutics leads to cancers developing drug resistance. Thus, Fearon et al. developed RTK drug resistant endometrial cancer cell lines in 3D cultures with known mutations of FGFR to explore the mechanism of developed resistance with 14 days of RTK inhibitor (RTKi) treatment. The phosphoproteomes of cells before, during, and after 14 days of the RTKi therapy revealed a significant change in phosphorylated proteins as treatment progressed, suggesting an adaptation of phosphorylated signaling pathways in response to this RTKi. In particular, Fearon et al. showed an increase of phosphorylated proteins involved in AKT signaling. A microarray that compared gene expression levels in the RTKi resistant cell line with that of its parental cell line revealed PHLDA1 as the most significantly downregulated gene in the RTKi resistant cells. The authors also reported that PHLDA1 expression levels are correlated with less susceptibility of cancer cells developing resistance to RTKi therapies. Ectopic expression of inducible full-length PHLDA1 in the RTKi resistant cancer cells re-sensitized the cells to RTKi therapy while ectopic expression of a PH domain mutant PHLDA1 did not re-sensitize cells to RTKi therapy. Furthering the scope of lower PHLDA1 expression levels attributed to RTKi therapy resistance, the authors studied another RTK-driven cancer, human epidermal growth factor receptor 2 (HER2) positive breast cancer. MCF7/HER2-18 cells were injected into SCID mice followed by bi-weekly treatment of a frontline RTKi therapeutic for HER2+ breast cancer, trastuzumab, or IgG control. PHLDA1 expression levels in remaining tumors in these mice were decreased compared to controls, again suggesting that chemoresistance is developed in cancer cells due to aberrantly activated AKT signaling upon downregulation of PHLDA1. Indeed this hypothesis was validated in two additional HER2+ breast cancer cell lines in a study similar to the RTKi resistant endometrial cancer cell line described above [12].

In conclusion, the PHLDA family of proteins may play both tumor suppressive and oncogenic roles in tumorigenesis though more studies are needed to decipher mechanisms underlying these differential roles. The work of Fearon et al. provides a novel mechanism for the PHLDA family, particularly PHLDA1, as generally being a global tumor suppressor by inhibiting AKT signaling as cancer cells undergo selective pressures from chemotherapeutics. How cancers alter the expression of the PHLDA family members under selective pressures of chemotherapeutics is an important question that remains to be addressed. Interestingly, a new class of noncoding RNAs, circular RNAs (circRNAs), can alter gene expression by acting as “sponges” of miRNAs and has been shown to increase PHLDA1 expression [25]. Additional research dedicated to the multiple strategies that cancers can utilize to alter the expression of PHLDA family members could offer more molecular insight into the development of chemoresistance. The importance of temporal expression of PHLDA family members may help explain their context-dependent roles in cancer progression or suppression. An example, PHLDA1 was shown to be highly expressed in salivary gland development followed by a sharp decline in expression during later developmental stages [26].

Contrast to the PHLDA family members, the PHLDB family members are understudied for their contribution to cancer. All published studies classify this family as oncogenes (Table 1). For example, genome-wide association studies (GWAS) linked single nucleotide polymorphisms (SNPs) of PHLDB1 with susceptibility to develop glioblastomas [21,27] and pituitary tumors [22]. Also, PHLDB2 is highly expressed in metastatic colorectal patients. Treatment of colorectal cancer cell line HCT116 null for p53 with growth factors responsible for epithelial-to-mesenchymal transition (EMT) resulted in an increased expression of PHLDB2 [28]. PHLDB2 then binds to MDM2 and aids in the degradation of E-cadherin which could contribute to metastatic colorectal cancer independent of p53 [28]. PHLDB2 contribution to metastasis fits with PHLDB2 proposed biological function of aiding microtubule anchoring to focal adhesion points necessary for cell motility [29]. PHLDB3 is the least studied protein in this family, as there has been only one published implication in development of non-small cell lung carcinoma (NSCLC) as a novel fusion protein with fibroblast growth factor receptor discovered in a single patient sample out of 26,054 NSCLC samples screened using a hybrid capture–based comprehensive genomic profiling [30].

Thus far, all the existing lines of evidence indicate that the PHLDB family members appear to play oncogenic roles in cancer development. Further systematic and in-depth studies are necessary to better understand the biological roles of these PHLDB proteins in cancer development and progression as well as their underlying mechanisms.

### p53 and the PHLD Class Proteins

**Figure 2:**
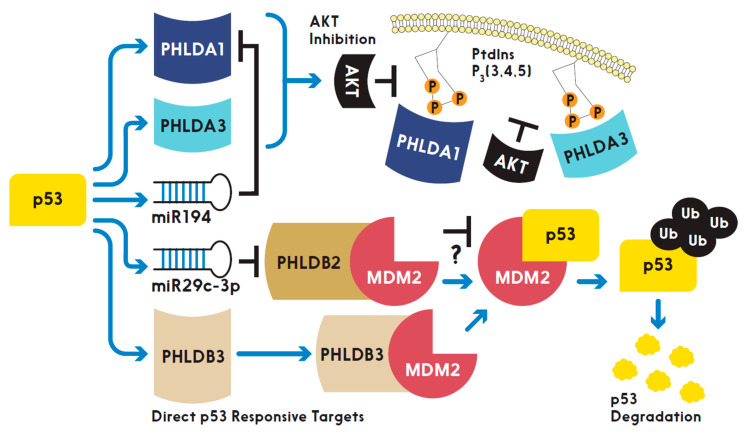
A schematic depicting the interplay between p53 and the PHLD class proteins in regulation of cell signaling. “T” shaped bars indicate “inhibition” while arrows denote “activation” or “promotion” and “?” suggests “unknown”.

PHLD class proteins have been suggested to be both direct and indirect targets of p53 at their transcriptional levels. Two research groups with overlapping researchers have established two of the three PHLDA family members as direct targets of p53. Figure 2 highlights the studied relationships between p53 and the PHLD class proteins. PHLDA3, the PH domain-only A family member, was found to be a direct transcriptional target of p53 by comparison of microarray data from γ-irradiation or 5-fluorouracil treated cells expressing wild type p53 or all serine to alanine substitutions in the transactivation-domain of p53 [13]. The induction of mRNA expression levels of PHLDA3 in cells with wild type p53 after treatment with DNA-damaging agents were like that of the canonical target of p53, p21. In addition, increased protein expression of PHLDA3, NOXA and p21 also correlated with activated p53. However, the closest homolog to PHLDA3, PHLDA2, was shown to not be a transcriptional target of activated p53. Furthering PHLDA3 as a direct transcriptional target of p53 was the presence of p53 responsive elements upstream of PHLDA3 transcriptional start site. Activated p53 bound to this responsive element as validated with a chromatin immunoprecipitation (ChIP) assay. Ultimately, PHLDA3 acted as a p53 responsive dominant negative inhibitor of AKT by outcompeting AKT binding to PtdI(3,4,5)P_3_ and PtdI(4,5)P_2_ at the membrane resulting in incomplete activation of AKT and thus PHLDA3 functions as a tumor suppressor [13]. As Kawase et al. highlighted, activated AKT phosphorylates MDM2 at serine 166 (P-Ser166) which increases degradation of p53 [13]. Indeed, Kawase et al., under basal conditions in LNCaP cells, observed endogenous PHLDA3 decreased activated AKT (via P-Ser473), decreased MDM2 P-Ser166, and increased p53 protein levels when compared to LNCaP cells treated with siRNA targeting PHLDA3. The choice of LNCaP cells was appropriate as these cells are known to harbor constitutively active AKT signaling [31]. More recently, the same research group revealed with microarray and ChIP assays that, like PHLDA3, PHLDA1 is a direct target of p53, functions as a p53 responsive dominant negative inhibitor of AKT and upregulation of PHLDA1 was not observed in p53 null cells [11]. However, they did not research a direct effect of PHLDA1 activity on MDM2 P-Ser166 and p53 expression [11]. Interestingly, the authors analyzed the TCGA database and found that human cancers expressing PHLDA1 have a total of 62 amino acids mutated and half of those mutations are located in the PH domain of PHLDA1 [11]. This suggests that the PH domains of PHLDA1 and PHLDA3 are necessary for their proposed biological function as tumor suppressors by inhibiting AKT activation as proposed by Fearon et al [12]. The authors also pointed out that many cancers analyzed express a shorter isoform of PHLDA1, however, the AKT inhibitory activity of the shorter isoform is equal to the longer isoform of PHLDA1. The existence of isoforms for the PHLDA family members, as the authors pointed out, should be distinguished when studying PHLDA1 in future functional assays [11]. Though not further elaborated on by the authors, shorter isoforms could exclude interacting partners experienced by the full-length isoform, which may alter PHLDA1 activity in various tissues expressing the shorter isoform. PHLDA1 was recently reported to be a target of miR-194 [9]. It is worth noting that miR194 is a known target of activated p53 [32]. Thus, a possible regulatory network may exist where activated p53 upregulates miR-194 to downregulate PHLDA1 expression levels but has yet to be directly researched.

Similar to the PHLDA family, the PHLDB family of proteins have also been shown as both direct and indirect targets of p53. Through analysis of the TCGA genome and Oncomine databases, we found that various cancers overexpress PHLDB3 and unraveled PHLDB3 as a potential oncogene by inactivating p53. Surprisingly, PHLDB3 enhanced p53 degradation through MDM2 activity in negative feedback fashion [24]. Ectopic PHLDB3 in cancer cells reduced the protein, but not mRNA, levels of p53. The degradation of p53 protein occurred when PHLDB3 interacting with MDM2 ubiquitinated p53. Treating cells with a proteasome inhibitor, MG132, reversed p53 degradation. Thus, dysregulation of PHLDB3 in human cancers could contribute to tumor growth despite these tumors having wild type p53 present. In addition, PHLDB3 may have p53-independent roles leading to cancer, as siRNA-mediated depletion of PHLDB3 in p53 null HCT116 cells also decreased cell proliferation. This observation may be attributed to PHLDB3 enhancing MDM2 to inhibit p53 homologs, such as TAp73, resulting in decreased expression of tumor suppressors independent of p53. Therefore, PHLDB3 is a direct target of activated p53, determined by observing increased mRNA levels of PHLDB3 in cells treated with DNA damaging agents along with ChIP analysis further validating that PHLDB3 is a direct p53 target as activated p53 bound to a p53 responsive element 4.8kb upstream of the PHLDB3 gene [24]. These observations were not found in a p53 null cell line. Different from PHLDB3, PHLDB2 was shown to be reduced at RNA levels in response to activated p53, as a p53 inducible miR-29c targeted PHLDB2 mRNA for degradation [23]. The genomic sequence that encodes miRNA-29c contained two upstream p53 response elements, though, the authors did not perform ChIP analysis of these proposed p53 response elements. Rather, they validated the p53 response elements by cloning the sequences into a luciferase reporter for transfection into a wild type p53 cell line followed by treatment with DNA damaging agents and monitoring of luciferase activity. Though direct validation of p53 bound to these proposed p53 response elements upstream of the miR-29c with ChIP assay would be preferred, this data suggests that PHLDB2 expression is indirectly responsive to activated p53 transcriptional activity. Interestingly, PHLDB2 was recently reported to bind to MDM2 [28]. Whether this interaction increases stability of p53 levels is of interest given that the study found PHLDB2 bound to MDM2 in a p53 null cell line [28]. PHLDB1 has not been studied in context of being a target for p53, though, attempts to find potential p53 responsive elements upstream of the PHLDB1 transcriptional start site should warrant research if PHLDB1 is indeed a target of p53 transcriptional activity. These studies as briefly reviewed in this and the above sections strongly suggest that both PHLDA and PHLDB families play roles in the regulation of the p53 pathway directly and indirectly, which is highly associated with cancer development.

## 3. Prospects and More Questions 

The PHLD class of proteins warrant more investigation to clarify their roles in human pathology and normal metabolic homeostasis. The common PH domain shared by all class members establishes a spatial restriction at cell membranes (via binding to phosphorylated lipids) where PHLD class proteins may carry out many of their functions. Careful interrogation of this spatially distinct region of the cell using techniques able to decipher PHLD interacting partners, such as mammalian membrane two-hybrid assay [33], can expand additional roles PHLD class proteins have on various signaling stimuli which may lead to pathological states. The presence of various isoforms for PHLD class proteins also need to be recognized when carrying out functional assays as Chen et al. highlighted [11]. Studying truncated isoforms of PHLD class proteins while maintaining the PH domain may exclude interacting partners particularly at the membrane. Therefore, both spatial location and expression levels of isoform should be considered when studying the PHLD class proteins.

A high-throughput molecular dynamic simulation of PH domain-containing proteins interacting at the membrane surface with PtdIns was recently established [34]. The simulations and the proposed analyses provide mechanistic insights for PHLD class proteins binding to PtdIns at the membrane. These insights could better inform possible conformational changes of PH domain-containing proteins upon lipid binding at the membrane, which may lead to plausible docking sites for other interacting partners. The authors note that a common protein fold is present for the majority of PH domain-containing proteins in addition to a common KXn(K/R)XR motif within the PH domain. The motif provides a positively charged region for electrostatic interactions with PtdIns at the membrane surface [34]. The PHLD class proteins all possess a version of the KXn(K/R)XR motif, however, with differences in the 4th and 5th positions of the motif. Interestingly, the PHLDA family has a more net positively charged KXn(K/R)XR motif than do the PHLDB family members, with all PHLDB family members possessing a large hydrophobic tryptophan residue in the 4th position of the motif. The PHLDA family members having a more positively charged motif within the PH domain versus the PHLDB family may provide more favorable interactions with PtdIns at the membrane surface. This could begin to explain the duality of activities reported for each family. Though, to model such interactions as outlined [34] would require the need for crystal structures to be solved for the PHLD class proteins, which currently is not available searching the Protein Data Bank (rcsb.org). However, PHLDB family members with a mutated KXn(K/R)XR motif to reflect that of the PHLDA family members’ positively charged motif could result in a gain-of-function for PHLDB family members, particularly with AKT inhibition.

In contrast to the PHLDA family, the PHLDB family potentiates AKT signaling. PHLDB1 overexpression enhances insulin signaling in cultured adipocytes by modulating AKT phosphorylation and recruitment of GLUT4 during insulin-dependent glucose uptake [35]. PHLDB1 overexpression also increased activation of p70S6K (via P-Thr389) which is a well-known downstream target of AKT enabling protein synthesis. However, these observations were not found with overexpression of PHLDB2 which suggests that the presence of the PH domain may not be necessary for PHLDB1 to enhance AKT activity in response to insulin given that PHLDB2 also contains a homologous PH domain. Rather, the presence of the FHA domain of PHLDB1 (which is absent in PHLDB2 and PHLDB3) may be critical for modulating insulin signaling by binding to newly phosphorylated proteins in response to insulin signaling. There are currently no published studies showing a role of PHLDB3 in metabolism like that of other PHLD class members. Increased AKT signaling is well established in its role to promote metabolism in cancer as previously reviewed [36]. Thus, the oncogenic roles observed for the PHLDB family may be a result of enhancing AKT signaling, resulting in an increase in metabolic state in cancers where PHLDB family members are overexpressed. The differential effects of the two families on AKT signaling requires further investigation. AKT signaling has multiple downstream effectors which aid tumor progression and metabolism, and thus understanding how the two families alter AKT signaling will impact their roles as either tumor suppressors or oncogenes. Several publications reviewed in this essay establish the PHLDA family as inhibitors of AKT activation in different cell types. However, only one study suggests that PHLDB1, not PHLDB2, enhances AKT signaling [35] with PHLDB3 effects on AKT signaling yet to be researched. In addition, PHLDB1 enhancing AKT activation theoretically should lead to p53 degradation via the AKT mediated phosphorylation of MDM2, further establishing PHLDB1 as an oncogene, though this possibility remains to be investigated in the future. As mentioned above, the duality of inhibition or activation of AKT between the two families may be the result in the lipid binding motif within the PH domain. Therefore, future efforts need to address if PHLDB3 modulates the AKT pathway and if this provides an advantage for tumor survival. The PHLDB family could function to counteract PHLDA family members inhibition of AKT. This potential crosstalk between the families may maintain normal AKT signaling, particularly at the membrane surface, as a well-maintained feedback loop. Research addressing PHLDB family proteins modulation of AKT signaling in more tissue types will undoubtedly provide the field with a function for the PHLDB family. 

In addition, further interrogation of the PHLDB3-MDM2 interaction is needed to address if PHLDB3 stabilizes MDM2 to bind with other adaptor proteins (such as MDMX) for increased ubiquitination of p53 or if PHLDB3 enhances MDM2 E3 ubiquitin ligase activity alone. The former scenario would establish PHLDB3 with homologous functions of MDMX as it pertains to enhancing MDM2 ubiquitination activity of p53. The various mechanisms of gene regulation that cancer cells may alter to influence expression of PHLD class proteins can both begin to explain differential expression levels of PHLD class proteins in tissue specific context and during tumor progression. Research designed to identify additional novel miRNAs silencing PHLD class protein expression and as mentioned previously, circRNAs, to counteract miRNA function [25] will establish regulatory networks that cancer cells can manipulate for survival. Unveiling these regulatory networks will classify subsets of cancers that are more tumorigenic than others. The use of PHLD knockout mice to model potential disease states in vivo is a powerful tool needed to expand PHLD class research. However, the use of knockout animals may require more than one PHLD family member knockout given that other homologous family members may provide a compensatory function to maintain homeostasis. In summary, more work is needed to fully elucidate the functions of the PHLD class, particularly the PHLDB family, for p53-dependent and independent roles in cancer and metabolism as well as for animal and organ development. Proper experimental considerations including cellular location and temporal expression of PHLD class proteins need to be applied when researching this class of proteins. The information gained from these studies on these molecules and their pathways would certainly be useful for future design strategies for target-specific therapies against cancers and metabolic disorders involving the PHLD class proteins.

## Figures and Tables

**Table 1:** PHLD Class Proteins and Cancer.

	Expression Level in Normal Tissues *	Expression Level in Disease	References
PHLDA1	All Tissues ↑ Salivary Glands	↓ Breast Cancer	[7,11,12,15]
		↓ Ovarian Cancer	[7,11]
		↓ Endometrial Cancer	[7,12]
		↓ Lung Cancer	[15]
		↓ Gastric Cancer	[7,16]
		↑ Colorectal Cancer	[7,17]
		↑ Osteosarcoma	[7,8]
PHLDA2	Many Tissues ↑ Placenta	↓ Breast Cancer	[14]
		↓ Lung Cancer	[14]
		↓ Osteosarcoma	[18]
PHLDA3	All Tissues	↓ Lung Cancer (Neuroendocrine Tumor)	[13]
		↓ Pancreatic Cancer (Neuroendocrine Tumor)	[19,20]
PHLDB1	Many Tissues ↑ Brain	SNPs ↑ Brain Cancer	[21,22]
PHLDB2	Many Tissues ↑ Placenta	↑ Colorectal Cancer	[23]
PHLDB3	All Tissues	↑ Colorectal Cancer	[24]

* RNA expression cited from the Human Protein Atlas (proteinatlas.org).

## References

[1] Haslam R.J., Koide H.B., Hemmings B.A. (1993). Pleckstrin domain homology. Nature.

[2] Lemmon M.A. (2007). Domains and Phosphoinositides. Biochem. Soc. Symp..

[3] Dowler S., Currie R.A., Campbell D.G., Deak M., Kular G., Downes C.P., Alessi D.R. (2000). Identification of pleckstrin-homology-domain-containing proteins with novel phosphoinositide-binding specificities. Biochem. J..

[4] Durocher D., Jackson S.P. (2002). The FHA domain. FEBS Lett..

[5] Park C.G., Lee S.Y., Kandala G., Lee S.Y., Choi Y. (1996). A Novel Gene Product that Couples TCR Signaling to Fas(CD95) Expression in Activation-Induced Cell Death. Immunity.

[6] Frank D., Mendelsohn C.L., Ciccone E., Svensson K., Ohlsson R., Tycko B. (1999). A novel pleckstrin homology-related gene family defined by Ipl/Tssc3, TDAG51, and Tih1: Tissue-specific expression, chromosomal location, and parental imprinting. Mamm. Genome.

[7] Nagai M.A. (2016). Pleckstrin homology-like domain, family A, member 1 (PHLDA1) and cancer. Biomed. Rep..

[8] Ren L., Mendoza A., Zhu J., Briggs J.W., Halsey C., Hong E.S., Burkett S.S., Morrow J.J., Lizardo M.M., Osborne T. (2015). Characterization of the metastatic phenotype of a panel of established osteosarcoma cells. Oncotarget.

[9] Liu L., Shi Y., Shi J., Wang H., Sheng Y., Jiang Q., Chen H., Li X., Dong J. (2019). The long non-coding RNA SNHG1 promotes glioma progression by competitively binding to miR-194 to regulate PHLDA1 expression. Cell Death Dis..

[10] Totzeck F., Andrade-Navarro M.A., Mier P. (2017). The Protein Structure Context of PolyQ Regions. PLOS ONE.

[11] Chen Y., Takikawa M., Tsutsumi S., Yamaguchi Y., Okabe A., Shimada M., Kawase T., Sada A., Ezawa I., Takano Y. (2018). PHLDA1, another PHLDA family protein that inhibits Akt. Cancer Sci..

[12] Fearon A.E., Carter E.P., Clayton N.S., Wilkes E.H., Baker A.-M., Kapitonova E., Bakhouche B.A., Tanner Y., Wang J., Gadaleta E. (2018). PHLDA1 Mediates Drug Resistance in Receptor Tyrosine Kinase-Driven Cancer. Cell Rep..

[13] Kawase T., Ohki R., Shibata T., Tsutsumi S., Kamimura N., Inazawa J., Ohta T., Ichikawa H., Aburatani H., Tashiro F. (2009). PH Domain-Only Protein PHLDA3 Is a p53-Regulated Repressor of Akt. Cell.

[14] Wang X., Li G., Koul S., Ohki R., Maurer M., Borczuk A., Halmos B. (2015). PHLDA2 is a key oncogene-induced negative feedback inhibitor of EGFR/ErbB2 signaling via interference with AKT signaling. Oncotarget.

[15] Li G., Wang X., Hibshoosh H., Jin C., Halmos B. (2014). Modulation of ErbB2 Blockade in ErbB2-Positive Cancers: The Role of ErbB2 Mutations and PHLDA1. PLOS ONE.

[16] Zhao P., Lu Y., Liu L. (2015). Correlation of decreased expression of PHLDA1 protein with malignant phenotype of gastric adenocarcinoma. Int. J. Clin. Exp. Pathol..

[17] Chiu S.-T., Hsieh F.-J., Chen S.-W., Shu H.-F., Li H. (2005). Clinicopathologic Correlation of Up-regulated Genes Identified Using cDNA Microarray and Real-time Reverse Transcription-PCR in Human Colorectal Cancer. Cancer Epidemiol. Biomark. Prev..

[18] Li Y., Song X., Liu Z., Li Q., Huang M., Su B., Mao Y., Wang Y., Mo W., Chen H. (2019). Upregulation of miR-214 Induced Radioresistance of Osteosarcoma by Targeting PHLDA2 via PI3K/Akt Signaling. Front. Oncol..

[19] Takikawa M., Ohki R. (2017). A vicious partnership between AKT and PHLDA3 to facilitate neuroendocrine tumors. Cancer Sci..

[20] Ohki R., Saito K., Chen Y., Kawase T., Hiraoka N., Saigawa R., Minegishi M., Aita Y., Yanai G., Shimizu H. (2014). PHLDA3 is a novel tumor suppressor of pancreatic neuroendocrine tumors. Proc. Natl. Acad. Sci. USA.

[21] Li X., Cao H., Liu Y. (2018). Genetic epidemiology and risk factors for brain tumors. J. Cent. South Univ. Med. Sci..

[22] Kim L.H., Kim J.-H., Namgoong S., Cheong H.S., Yoon S.-J., Kim E.H., Kim S.H., Kim S.H., Chang J.H., Shin H.D. (2019). A PHLDB1 variant associated with the nonfunctional pituitary adenoma. J. Neuro-Oncology.

[23] Chen G., Zhou T., Li Y., Yu Z., Sun L. (2017). p53 target miR-29c-3p suppresses colon cancer cell invasion and migration through inhibition of PHLDB2. Biochem. Biophys. Res. Commun..

[24] Chao T., Zhou X., Cao B., Liao P., Liu H., Chen Y., Park H.-W., Zeng S.X., Lu H. (2016). Pleckstrin homology domain-containing protein PHLDB3 supports cancer growth via a negative feedback loop involving p53. Nat. Commun..

[25] Wang K.-W., Dong M. (2019). Role of circular RNAs in gastric cancer: Recent advances and prospects. World J. Gastrointest. Oncol..

[26] Gomes A.N.D.M., Nagai M.A., Lourenço S.V., Coutinho-Camillo C.M. (2019). Apoptosis and proliferation during human salivary gland development. J. Anat..

[27] Viana-Pereira M., Moreno D.A., Linhares P., Amorim J., Nabiço R., Costa S., Vaz R., Reis R.M. (2019). Replication of GWAS identifies RTEL1, CDKN2A/B, and PHLDB1 SNPs as risk factors in Portuguese gliomas patients. Mol. Boil. Rep..

[28] Chen G., Zhou T., Ma T., Cao T., Yu Z. (2019). Oncogenic effect of PHLDB2 is associated with epithelial-mesenchymal transition and E-cadherin regulation in colorectal cancer. Cancer Cell Int..

[29] Seetharaman S., Etienne-Manneville S. (2019). Microtubules at focal adhesions - a double-edged sword. J. Cell Sci..

[30] Qin A., Johnson A., Ross J.S., Miller V.A., Ali S.M., Schrock A.B., Gadgeel S.M. (2019). Detection of Known and Novel FGFR Fusions in Non–Small Cell Lung Cancer by Comprehensive Genomic Profiling. J. Thorac. Oncol..

[31] Nesterov A., Lu X., Johnson M., Miller G.J., Ivashchenko Y., Kraft A.S. (2001). Elevated Akt Activity Protects the Prostate Cancer Cell Line LNCaP from TRAIL-induced Apoptosis. J. Boil. Chem..

[32] Braun C.J., Zhang X., Savelyeva I., Wolff S., Moll U.M., Schepeler T., Ørntoft T.F., Andersen C.L., Dobbelstein M. (2008). p53-Responsive micrornas 192 and 215 are capable of inducing cell cycle arrest. Cancer Res..

[33] Saraon P., Grozavu I., Lim S.H., Snider J., Yao Z., Stagljar I. (2017). Detecting Membrane Protein-protein Interactions Using the Mammalian Membrane Two-hybrid (MaMTH) Assay. Curr. Protoc. Chem. Boil..

[34] Yamamoto E., Kalli A.C., Yasuoka K., Sansom M.S. (2016). Interactions of Pleckstrin Homology Domains with Membranes: Adding Back the Bilayer via High-Throughput Molecular Dynamics. Struct..

[35] Zhou Q.L., Jiang Z.Y., Mabardy A.S., Del Campo C.M., Lambright D.G., Holik J., Fogarty K.E., Straubhaar J., Nicoloro S., Chawla A. (2010). A Novel Pleckstrin Homology Domain-containing Protein Enhances Insulin-stimulated Akt Phosphorylation and GLUT4 Translocation in Adipocytes*. J. Boil. Chem..

[36] Robey R.B., Hay N. (2008). Is Akt the “Warburg kinase”?—Akt-energy metabolism interactions and oncogenesis. Semin. Cancer Boil..

